# Development and Validation of Liquid Chromatography-Tandem Mass Spectrometry Method for Simple Analysis of Sumatriptan and its Application in Bioequivalence Study

**DOI:** 10.3390/ph13020021

**Published:** 2020-01-24

**Authors:** Wisut Wichitnithad, Siriwan Nantaphol, Petploy Vicheantawatchai, Thanyaporn Kiatkumjorn, Wachirasak Wangkangwan, Pornchai Rojsitthisak

**Affiliations:** 1 Department of Bioanalytical Development, Pharma Nueva Co., Ltd., Bangkok 10900, Thailand; wisutrd@yahoo.com (W.W.); siriwan.nantaphol@pharmanueva.com (S.N.); petploy.vicheanta@pharmanueva.com (P.V.); thanyaporn.kiatkumjorn@pharmanueva.com (T.K.); 2 Natural Products for Ageing and Chronic Diseases Research Unit, Chulalongkorn University, Bangkok 10330, Thailand; 3 Department of Clinical Development, Pharma Nueva Co., Ltd., Bangkok 10900, Thailand; wachirasak.wangkangwan@pharmanueva.com; 4 Department of Food and Pharmaceutical Chemistry, Faculty of Pharmaceutical Sciences, Chulalongkorn University, Bangkok 10330, Thailand

**Keywords:** sumatriptan, LC-MS/MS, liquid-liquid extraction, pharmacokinetics, bioequivalence

## Abstract

This work demonstrated a sensitive, selective, and simple liquid chromatography-tandem mass spectrometry (LC-MS/MS) method for quantitation of sumatriptan in human plasma samples. Terazosin was used as an internal standard to minimize the variability during sample processing and detection. Sample cleanup prior to chromatographic analysis was accomplished by liquid-liquid extraction (LLE) with *tert*-butyl methyl ether (*t*-BME). The separation was performed on a reversed-phase Symmetry^®^ C18 column (150 × 4.6 mm i.d., 5 µm) under a gradient mode, using a 0.2% formic acid aqueous solution and acetonitrile at a flow rate of 0.5 mL/min. Sumatriptan (*m/z* 296.26→251.05) and terazosin (m/z 388.10→290.25) were quantified using a triple quadrupole mass spectrometer, operating in the multiple reaction monitoring (MRM) under the positive ion mode. The method was fully validated following US-FDA and EMA guidelines. The LC-MS/MS assay had a calibration range of 0.5–50.0 ng/mL. The assay was precise and accurate with a between-run precision of <9.51%, and between-run accuracy between −7.27 to 8.30%. The developed method was subsequently applied in the determination of plasma concentration-time profile of a sumatriptan 50-mg tablet following oral administration in healthy volunteers.

## 1. Introduction

Sumatriptan is a drug for migraine and cluster headaches treatment [1,2]. It is a selective 5-hydroxytryptamine (5-HT_1B/1D_) receptor agonist. The binding between sumatriptan and serotonin type-1D receptors results in vasoconstriction of extensively dilated cranial blood vessels and subsequent relief of migraine-related pain. Sumatriptan is commercially available in several dosage forms such as oral, nasal, and parenteral delivery products. The recommended dose is a single 50 mg tablet with the maximum daily dose of 100 mg. The pharmacokinetics of sumatriptan depend on the route of administration, in which the most common route is oral administration. Oral bioavailability of sumatriptan is low (approximately 14%) because of extensive hepatic first-pass metabolism and incomplete absorption. After oral administration of a sumatriptan 50 mg tablet, it shows *t*_max_ at 1.13 h with *C*_max_ of 33.21 ng/mL and an elimination half-life (*t*_1/2_) of 2.96 h [3].

Several analytical techniques have been developed for the quantitative determination of sumatriptan, including voltammetry [4,5], ultraviolet (UV) spectroscopy [6], and liquid chromatography (LC) coupled with UV [7,8,9], fluorescence [10], electrochemical [11,12], and mass spectrometric (MS) detectors [3,13,14,15,16]. Electrochemical, UV–vis, and fluorescent spectroscopic techniques can be advantageous as they are fast, simple, and require inexpensive instruments. However, these methods are limited by sensitivity and/or specificity and often require a large amount of sample, which is unsuitable for analyzing trace amounts of sumatriptan in human serum samples [4,5,6,10,11,12]. Liquid chromatography-tandem mass spectrometry (LC-MS/MS) has been demonstrated as a routine technique for applications in pharmaceutical analyses that affords high throughput, good sensitivity, and selectivity. A few LC-MS/MS methods were developed to quantify sumatriptan in human plasma. For example, Stewart et al. described an LC/ESI-MS/MS method for the simultaneous determination of sumatriptan, naratriptan, zolmitriptan, and rizatriptan in human serum using a solid-phase extraction (SPE) for sample preparation and bufotenine as an internal standard (IS). This method exhibited a linear range of 1–100 ng/mL with a lower limit of quantification (LLOQ) of 250 pg/mL [16]. Patel et al. developed ultra-performance liquid chromatography–tandem mass spectrometry with SPE for the simultaneous determination of sumatriptan and naproxen in human plasma using naratriptan and indomethacin as an IS. The linear range was validated from 0.050 to 100 ng/mL [14].

Besides the selection of an analytical technique, sample preparation is another key factor for quantitative analysis of sumatriptan in human plasma samples because the complexity of biological matrices can significantly affect accuracy and precision. This step must ensure an efficient extraction of the target analytes, adequate sample clean-up, and high sample throughput. Different extraction procedures have been used and the most common method for extracting sumatriptan is SPE [13,14,15,17]. SPE provides clean samples with good analyte recovery, but it usually requires complicated procedures and long sample preparation times, which may cause unintentional errors in the analytical results. Automated SPE systems with 96-well SPE cartridges were developed to overcome these limitations. These automated systems provide high throughput, accurate sample processing, and a reduction of labor cost. Liquid-liquid extraction (LLE) is an alternative technique for sample clean-up. This methodology is simple and robust but requires a skilled researcher. The consideration of an appropriate extraction solvent for LLE is important for achieving recovery, sufficiently clean sample, and high sample throughput. Ethyl acetate was used as a solvent for extracting sumatriptan in human plasma [3,10]. However, the use of ethyl acetate consumes time in an evaporation step (about 40 min per run). Ethyl acetate often extracts endogenous compounds from the sample matrix due to its polarity, which frequently results in a high background as well as ionization suppression in mass spectrometric detection. Therefore, the development of a simple, rapid, cost-effective, and sensitive method for the quantification of sumatriptan in human plasma is still in demand.

In this study, we demonstrated an LC-MS/MS with liquid-liquid extraction for the quantitative determination of sumatriptan in human plasma. *tert*-Butyl methyl ether (*t*-BME) was selected as an extraction solvent since it offers advantages over ethyl acetate and other organic solvents in terms of volatility and low water solubility. These properties allow it to yield high recovery and high selectivity with less evaporating time. The LC-MS/MS method was fully validated covering all validation parameters listed in the US-FDA and EMA guidelines on bioanalytical method validation [18,19] and then applied in pilot pharmacokinetic and bioequivalence studies of sumatriptan tablets.

## 2. Results and Discussion

### 2.1. Method Development

Matrix components in human plasma have been shown to be a source of variability and inaccuracy in LC-MS/MS analysis. Co-elution of endogenous phospholipids with an analyte can cause ion suppression or enhancement that also dramatically impact on quantitative LC-MS/MS. Therefore, the development of efficient sample preparation protocol to remove unwanted plasma matrix components and to selectively extract the compounds of interest is an essential part in bioanalysis. The common sample preparation techniques including protein precipitation, SPE, and LLE were taken into this consideration. Protein precipitation is a fast and simple protocol. However, this technique usually fails to sufficiently minimize endogenous interference. SPE allows removal of interfering biological matrix components and enhancing the concentrations of analytes. Unfortunately, complicated steps of SPE require some skill sets and well-training of the operator. Otherwise, this may lead to some unintentional errors and unreproducible results. In addition, the cost of disposable cartridges may limit the routine analysis in clinical settings. In this work, LLE with *t*-BME in comparison with other organic solvents was demonstrated as an effective solvent. *t*-BME is superior to the published extraction solvents e.g., ethyl acetate, hexane, and diethyl ether. The properties of this solvent allow the method to yield acceptable recovery and high selectivity with less evaporating time. For other solvents, for example, ethyl acetate, due to its more polarity compared to *t*-BME may often result in the contamination of more endogenous compounds from the sample matrix. However, high vapor pressure of diethyl ether and hexane make them hard in handling and volumetric transferring after extraction. Our developed method is advantageous for producing clean samples with sufficient efficiency as well as simple operation steps, which are more preferable for routine analysis.

**Figure 1:**
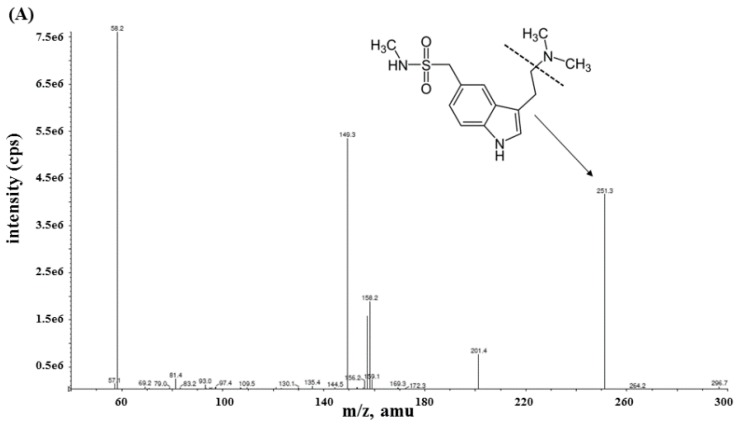

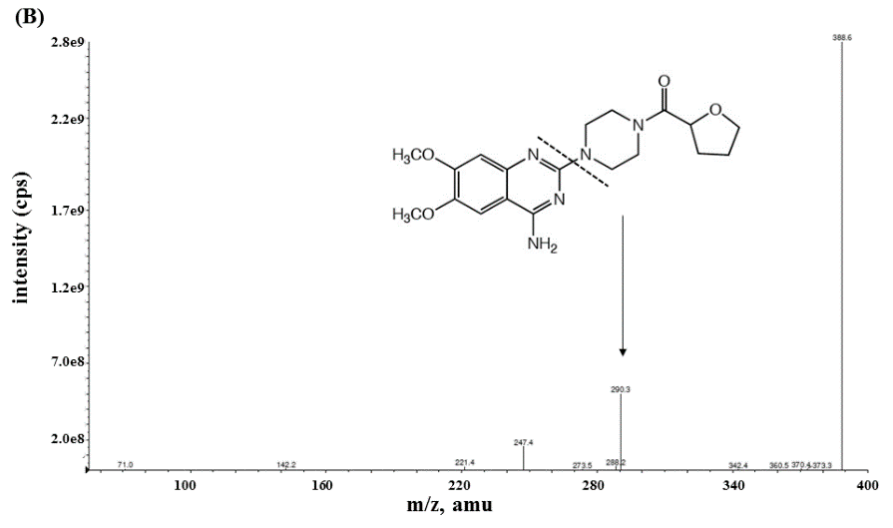
MS/MS spectra of (**A**) sumatriptan and (**B**) terazosin (**IS**).

The LC–MS/MS method for the determination of sumatriptan in human plasma was developed to achieve a rapid, selective, and sensitive method. The consideration of a suitable IS is an important factor to achieve accuracy and precision of quantitative analysis. Terazosin was appropriately chosen as an IS since it is stable in plasma and reproducible in LC-MS/MS system. Moreover, we found that sumatriptan and terazosin exhibited similar LC-MS/MS behaviors. The fragmentation mass spectra of sumatriptan and terazosin are shown in Figure 1. The precursor ions of sumatriptan and terazosin were found at *m/z* [M+H]^+^ 296.26 and 388.10, respectively. After fragmentation, the stable product ion of sumatriptan was observed at *m/z* 251.05 due to the elimination of dimethyl amine group. For terazosin, the stable product ion was observed at *m/z* 290.25 with the loss of piperazine tetrahydrofuran ketone side chain. Therefore, the appropriate multiple reaction monitoring (MRM) transitions of *m/z* 296.26→251.05 and m/z 388.10→290.25 were selected and also found to be specific for the detection of sumatriptan and terazosin, respectively. The chromatographic conditions were optimized to achieve good resolution, symmetric peak shape as well as a short run time. We found that mobile phase comprising of 0.2% formic acid aqueous solution (A) and acetonitrile (B) with the gradient program reported was the best condition. It provides fast analysis with retention time of 2.04 min for both sumatriptan and terazosin.

**Figure 2:**
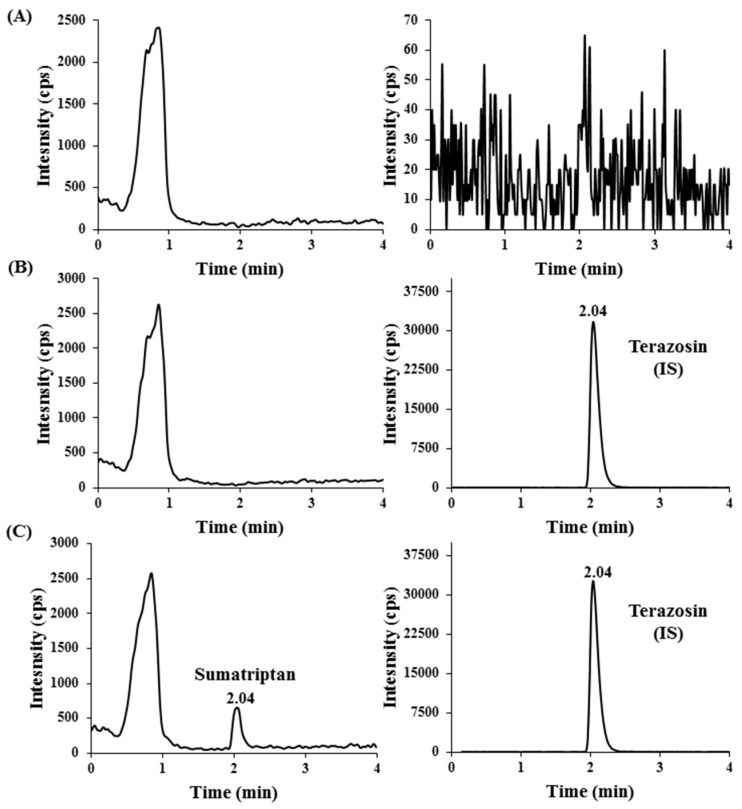
Representative chromatograms of (**A**) blank human plasma, (**B**) blank human plasma spiked with terazosin IS (41.3 ng/mL), and (**C**) human plasma spiked with Sumatriptan at the lower limit of quantification (LLOQ) (0.5 ng/mL) and terazosin IS (41.3 ng/mL). Left panel = sumatriptan; right panel = terazosin IS.

LLOQ is the minimum concentration at which the analyte could be quantified with acceptable accuracy and precision. The LLOQ level for the assay was 0.5 ng/mL. A representative chromatogram of an LLOQ sample is shown in Figure 2. The mean signal to noise ratio (S/N) was 8.83, which was >5 and met the required criterion. The %deviation was found in the range of −6.40 to 9.60 with %CV of 5.40–7.29. The large peak observed in ion chromatograms at the sumatriptan mass transition was excluded from the calculation of S/N ratio.

### 2.2. Method Validation

#### 2.2.1. Specificity and Sensitivity

The selectivity of the method from endogenous plasma components was investigated by analyzing eight different sets of blank human plasma. Figure 2 shows representative chromatograms of blank human plasma, blank plasma spiked with IS, and blank plasma spiked with IS and sumatriptan at LLOQ. No co-eluting peak that was >20% of the sumatriptan peak area at LLOQ level and no co-eluting compound with peak area >5% of the terazosin peak area were observed. The absence of interfering components in the chromatograms for sumatriptan and terazosin demonstrated assay selectivity in the matrix.

#### 2.2.2. Calibration Curves and Linearity

The linearity was tested by assessing signal responses of sumatriptan in human plasma at a concentration range of 0.5 to 50.0 ng/mL. All standard solutions contained the terazosin at 41.3 ng/mL. Calibration curves were constructed by plotting the concentrations of sumatriptan against peak area ratios of sumatriptan and terazosin. The calibration line was evaluated using weighted-linear least square model with a weighting factor of 1/x^2^. The analytical procedure was linear over the concentration range with a coefficient of determination (*r*^2^) of >0.99. The %deviations of at each calibration levels ranged from −6.01 to 2.27 with a %CV of 0.61 to 3.94. The results of calibration curves are summarized in Table 1.

#### 2.2.3. Accuracy and Precision

The within-run and between-run accuracy and precision were evaluated at five quality control (QC) levels. The results showed good accuracy and precision of the proposed method (Table 2). All the QC samples for within-run accuracy and precision exhibited %deviation in the range of −10.38 to 3.23 with %CV ranged from 2.15 to 5.40. For between-run accuracy and precision, %deviation varied from −7.27 to 8.30 and %CV was within 5.05 to 9.51.

#### 2.2.4. Extraction Recovery

The extraction recoveries of sumatriptan and terazosin were evaluated, in five replicates, by comparing the peak responses of pre-spiked low QC (LQC), medium QC (MQC), and high QC (HQC) samples to those of post-spiked samples. The average recoveries of extraction of sumatriptan at LQC, MQC, and HQC levels were 69.09, 72.73, and 74.27%, respectively, with %CV of 8.61, 9.47, and 8.61%, respectively. The average recovery of extraction of terazosin at 41.3 ng/mL was 38.72% with %CV of 4.79%. The %recovery precision of both sumatriptan and terazosin were within 15 %CV, indicating that the extraction efficiency of this method was adequate and reproducible. This demonstrates that the developed method has an acceptable extraction recovery of both sumatriptan and terazosin.

#### 2.2.5. Matrix, Hemolytic, and Lipemic Effects

The matrix effect on the detection of sumatriptan was determined at LQC and HQC concentrations. The results are summarized in Table 3. Although LLE could provide the more cost-effective sample preparation, the matrix effect can be observed in mass spectrometric analysis [20,21]. The IS-normalized matrix factor (MF) results indicated that there were some leftover matrix ions in the samples, leading to the ion suppression. However, the %CVs of the IS-normalized MF at LQC and HQC samples in nine lots of blank plasma were 5.99 and 1.89 respectively, suggesting that ion suppression did not have detrimental effects on the assay. Therefore, the developed method exhibited acceptable recovery with excellent accuracy and precision. However, the %CVs of the IS-normalized MF at LQC and HQC samples in nine lots of blank plasma were 5.99 and 1.89, respectively, indicating that ion suppression did not have detrimental effects on the assay. The effect of hemolytic on the analysis of sumatriptan is shown in Table 4. The average %deviation of LQC and HQC were −14.62 and −3.11, respectively, with %CV of 6.33 and 2.34%, respectively. The effect of lipid was also evaluated as shown in Table 4. The average %deviation of LQC and HQC were −14.67 and −4.29, respectively, with %CV of 4.26 and 3.65%, respectively. The %deviation and %CV were within the acceptance criteria, suggesting that hemoglobin components and lipoprotein particles in plasma did not cause any negative impacts on the proposed method.

#### 2.2.6. Dilution Integrity

Dilution linearity was evaluated in case that study sample concentrations were above the upper limit of quantification (ULOQ). Six replicates were performed for each diluted sample. The average %deviations for the back-calculated concentration of the dilution integrity sample of sumatriptan with 1:1 and 1:3 dilutions were 101.78 and 111.81% with %CV of 3.25 and 3.47%, respectively, indicating that up to 3-fold proportional dilution of human plasma samples is acceptable.

#### 2.2.7. Stability

The stability of sumatriptan in human plasma and stock solution was tested under different storage conditions. The results are presented in Table 5. The stock standard solutions of sumatriptan was stable in a refrigerator at 2–8 °C for 30 days and was stable at controlled room temperature (25 ± 5 °C) for 8 h. Sumatriptan in human plasma was stable for four freeze–thaw cycles, for at least 4 h in human plasma at room temperature (25 °C), for 77 days at −70 °C. The post-preparative stability under different storage conditions including dry state after extraction, autosampler, and reinjection was also studied. It was found that sumatriptan was stable for 2 days in dry state after extraction (stored in a refrigerator at 2–8 °C), and for 48 h in the autosampler (4 °C). Additionally, the sample kept in the autosampler (4 °C) can be reinjected within 48 h after the first injection. These results suggest that sumatriptan was stable for application in the routine analysis.

### 2.3. Method Application for Pharmacokinetic Study

**Figure 3:**
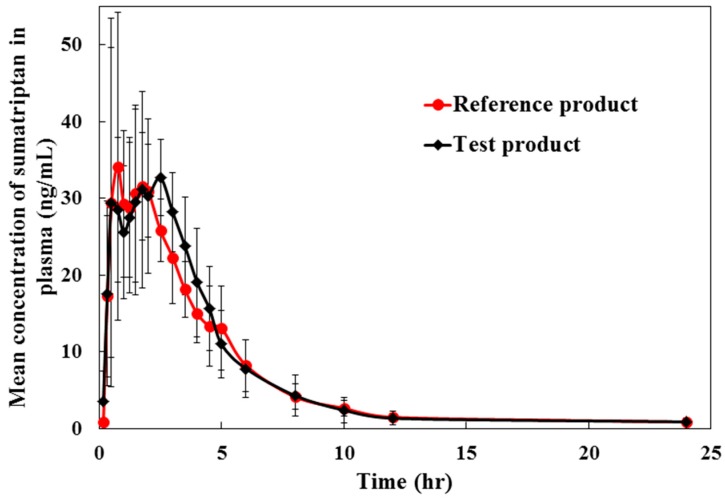
Mean plasma sumatriptan concentration-time curve after oral administration of a single dose of 50 mg of the test product (Generic sumatriptan 50 mg film-coated tablet; Siam Bheasach Co., Ltd., Bangkok, Thailand) and the reference product (Imigran^TM^ tablets 50 mg; GlaxoSmithKline, Poznan, Poland) (*n* = 6).

The validated method was applied to quantify sumatriptan in human plasma for the purpose of establishing the pilot bioequivalence study. Figure 3 shows the profiles of mean plasma concentrations versus time in six healthy subjects after a single oral administration of 50-mg sumatriptan tablets of the test and reference formulations under fasting condition. The values for the pharmacokinetic parameters and the geometric mean ratios of maximum plasma concentration (*C*_max_), area under the curve (AUC) from zero to last sampling time (AUC_0→t_), and AUC from zero extrapolated to infinite time (AUC_0→__∞_) of sumatriptan for the test drug as well as the reference drug are summarized in Table 6. The statistical analysis obtained showed that the point estimation of the geometric mean ratio (test/reference or T/R) of *C*_max_, AUC_0→t_, and AUC_0→__∞_ were within the equivalence criteria (80.00–125.00%) which were 103.88% (87.18–123.78%) for *C*_max_ ratio, 102.91% (92.68–114.26%) for AUC_0→t_ ratio, and 101.91% (90.47–114.78%) for AUC_0→__∞_ ratio. It can be concluded that these two sumatriptan film-coated tablet formulations established bioequivalence in terms of the rate and the extent of drug absorption. Additionally, the %different in incurred samples was within ±20% of the initial results, demonstrating the assay reproducibility during study.

## 3. Materials and Methods

### 3.1. Drugs and Chemicals

Sumatriptan succinate (lot no. G0L156, purity 98.4%) and terazosin hydrochloride dehydrate (lot no. G0F290, purity 91.9%) were purchased from U.S. Pharmacopeia (Rockville, MD, USA). The generic sumatriptan 50-mg film-coated tablets (Siam Bheasach Co., Ltd., Bangkok, Thailand) and Imigran^TM^ 50-mg tablets (GlaxoSmithKline Co., Ltd., Poznan, Poland) were used as the test and reference products, respectively. Acetonitrile, methanol and *t*-BME were purchased from Scharlau (Barcelona, Spain). Formic acid and 30% ammonia solution were obtained from Carlo Erba (Milano, Italy). All reagents were of at least analytical grade and used as received. Human blank plasma containing lithium heparin as an anticoagulant was obtained from IBS Co., Ltd. (Nakhon Pathom, Thailand). The purified water (18.2 MΩ cm) used throughout this experiment was obtained from a Milli-Q water purification system (Millipore; S.A.S, France).

### 3.2. Instrument Conditions

The liquid chromatographic system performed on a Prominence LC system (Shimadzu, Japan) consisted of a system controller (CBM-20Alite), a DGU-20A_3_ degasser, LC-20AD solvent delivery units, an FCV-11AL valve unit, a SIL-20AC autosampler, and a CTO-20A column oven. An analytical separation was carried out using a reversed-phase Symmetry^®^ C18 column (150 × 4.6 mm i.d., 5 µm) (Thermo Scientific, Tewksbury, MA, USA) placed in a CTO-20A column oven whose temperature was set at 35 °C. The elution of sample was performed in a gradient mode using a 0.2% formic acid aqueous solution (solvent A) and acetonitrile (solvent B) as a mobile phase at a flow rate of 0.5 mL/min. Gradient elution program was as follows: 0.00–0.02 min, B: 40%; 0.02–0.50 min, B: 40%; 0.50–1.00 min, B: 80%; 1.00–2.00 min, B: 80%; 2.00–2.20 min, B: 40%; 2.20–4.00 min, B: 40%. The injection volume was 10 µL. The total run time was 4 min. The entire flow was directed into the detector. Carry-over was evaluated by injecting an extracted blank sample after an upper limit of quantification (ULOQ) sample. For the acceptance criteria of the carry-over experiment, the response for the analyte and IS in a blank sample after the injection of a ULOQ standard must be lower than 20% and 5% of the analyte response in the LLOQ standard, respectively. In this study, there was no peak at the retention time of sumatriptan and terazosin presented in blank sample queued after ULOQ injection, suggesting that no carry-over effect was observed.

Mass spectrometric analysis of sumatriptan and terazosin (used as an IS) was achieved with MS/MS detection in a positive ion mode using an AB Sciex API-4000 mass spectrometer (Foster City, CA, USA) equipped with a Turbo ionspray™ interface which was set at 500 °C. The ion spray voltage was set at 4500 V. The source parameters including the collision gas, curtain gas, ion source gas 1 and ion source gas 2 were set at 4, 20, 50, and 50 psi, respectively. Nitrogen was designed as carrier and fragmentation gas. The compound parameters including the declustering potential (DP), entrance potential (EP), collision energy (CE), and collision cell exit potential (CXP) were 61, 10, 25, and 24 V for sumatriptan, respectively, and 116, 10, 35, and 24 V for terazosin, respectively. Detection of the ions was carried out in the multiple reaction monitoring mode (MRM) by monitoring the transition at *m/z* [M+H]^+^ 296.26**→**251.05 for sumatriptan and *m/z* [M’+H]^+^ 388.10**→**290.25 for terazosin with a 200 ms scan dwell time for all compounds. Quantitation of sumatriptan was based on the peak area ratio of sumatriptan versus terazosin. The data acquisition was processed using Analyst software™ (version 1.4.2). The calculations of validation data and plasma concentration were performed by Microsoft Excel program (Microsoft, Albuquerque, NM, USA).

### 3.3. Preparation of Stock Standard Solutions, Calibration Standards, and QC Samples

Stock standard solutions of sumatriptan and terazosin were prepared separately at 1 mg/mL in methanol. Working standard solutions of sumatriptan for calibration standards and QC samples were prepared from the 1 mg/mL sumatriptan stock solution. A working standard solution of terazosin at a concentration of 868.0 ng/mL was prepared from the stock solution of terazosin. All working solutions were diluted with a mixture of 0.2% formic acid solution and acetonitrile (60:40, *v/v*).

Seven-point calibration standard samples were prepared by spiking the working standard solutions in blank human plasma to yield final concentrations of 0.5 (LLOQ), 1.0, 2.5, 10.0, 20.0, 35.0, and 50.0 (ULOQ) ng/mL. Four concentration levels of spiked plasma QC samples were prepared at final concentrations of 0.5 (LLOQ QC), 1.5 (LQC), 25.0 (MQC), and 45.0 (HQC) ng/mL with 41.3 ng/mL of terazosin.

### 3.4. Sample Preparation

A 300 µL of plasma sample was transferred into a centrifuge tube. A 15 µL of terazosin working solution and 100 µL of 9.3 mol/L ammonium hydroxide solution were added to the plasma sample. The mixture was vortexed for 30 s followed by the addition of 2.0 mL of *t*-BME. The mixture was vortexed for 1 min and centrifuged at 1792 g, 4 °C for 10 min to make completely separated layers. A 1.7 mL of the upper layer was taken into a 2 mL microcentrifuge tube, and then evaporated to dryness at 45 °C for 10 min. After that, the residue was reconstituted with a 400 µL of a mixture of 0.2% formic acid solution and acetonitrile (60:40, *v/v*), followed by vortex-mixing for 30 s and centrifugation at 11,200 g, 4 °C for 10 min to remove undissolved matrix. The clear supernatant was taken out for LC-MS/MS analysis.

### 3.5. Method Validation

The method performance was evaluated according to US-FDA and EMA guidance for bioanalytical method validation for specificity, carry-over, linearity, accuracy, precision, LLOQ, recovery, dilution integrity, matrix effect, hemolytic and lipemic effect, and stability [18,19].

#### 3.5.1. Specificity and Sensitivity

Specificity of the method was performed using eight different lots of blank plasma (six lots of normal blank plasma, one lot of hemolyzed blank plasma, and one lot of hyperlipidemic plasma). The spiked samples at LLOQ were extracted and the presence or absence of interfering peak at the same retention time of sumatriptan or terazosin was investigated. Chromatograms from each blank sample were compared with the LLOQ sample.

The sensitivity of the method was evaluated at LLOQ concentration. The LLOQ is accepted if the sumatriptan response at LLOQ level is at least five times over the baseline response with a %deviation of ±20% and a percentage coefficient of variation (%CV) of ≤20%.

#### 3.5.2. Calibration Curve and Linearity

Calibration curves were evaluated from the analysis of spiked calibration samples at seven different concentration levels. The calibration curves were constructed by plotting the ratio of sumatriptan signal to IS signal as a function of sumatriptan concentrations. The equation model was obtained by weighted least squares linear regression analysis with a weighting factor of 1/x^2^. Back-calculation of the calibration standard concentration was used to confirm the suitability of equation. Each back-calculated concentration standard should be within ±15 %deviation, except for LLOQ which should be within ±20 %deviation. The coefficient of determination (*r*^2^) should be >0.99.

#### 3.5.3. Accuracy and Precision

Accuracy and precision were evaluated by analyzing drug concentration in plasma samples at LLOQ, LQC, MQC, HQC, and ULOQ levels. Six replicates of each concentration level were analyzed on the same day for intra-day (within-run) analysis and on three different days for inter-day (between-run) analysis. Accuracy was assessed by calculating the %deviation based on the difference between the mean concentration found and concentration added. The mean accuracy of each level should be within ±15 %deviation, except for LLOQ which should be within ±20%. Precision was evaluated by calculating the %CV of the mean concentration found. The %CV of each level should be within 15%, except for LLOQ which should not be more than 20%.

#### 3.5.4. Recovery

The recovery of extraction was evaluated from responses of sumatriptan obtained from QC samples in comparison with those obtained from post-spiked QC samples at the same concentration. The extraction recovery of sumatriptan was determined at LQC, MQC, and HQC levels. The extraction recovery of terazosin was determined at 41.3 ng/mL from MQC samples using the same procedure. The extents of recovery of analyte and IS should be consistent and reproducible.

#### 3.5.5. Dilution Integrity

Dilution integrity was evaluated to ensure that sample dilution with the same matrix did not have any effects on the reliability of the method. The accuracy and precision on 1:1 and 1:3 ratios (70.0 and 100.0 ng/mL, respectively) were analyzed. Samples at 70.0 ng/mL and 100.0 ng/mL of sumatriptan were diluted with human blank plasma to get diluted samples before analysis. The average %deviation should be ±15% of the actual concentration and %CV should be within 15%.

#### 3.5.6. Matrix Effect

The effect of extracted plasma matrix was evaluated in nine different sets of QC samples prepared from six different lots of human blank plasma, one lot of human hemolyzed blank plasma, one lot of human hyperlipidemic blank plasma, and one lot of human blank plasma containing 5.0 ng/mL of chlorpheniramine as a possible concomitant drug in case of drug allergic accident during study. The responses of sumatriptan obtained from post-spiked QC samples were compared with those obtained from the pure authentic standard solution at the same concentration. Each set of QC samples composed of three replicates of LQC and HQC samples. The matrix effect on terazosin was determined at 41.3 ng/mL. The effect of plasma matrix was separately determined for sumatriptan and terazosin and reported as MF. The ratio between the MF of sumatriptan and that of terazosin is calculated in term of the IS-normalized MF. The CV of IS-normalized matrix factor at each concentration level should be within 15%.

#### 3.5.7. Hemolytic and Lipemic Effects

The hemolytic and lipemic effects were evaluated by analyzing six replicates of samples containing sumatriptan at LQC (1.5 ng/mL) and HQC (45.0 ng/mL) levels in hemolyzed plasma and hyperlipidemic plasma, respectively. The concentrations of drug in plasma samples were determined against calibration curve prepared in human normal blank plasma. The acceptable limit of both effects should be within ±15 %deviation and 15 %CV.

#### 3.5.8. Stability

Stability experiments were performed with LQC and HQC samples under different conditions. The experiments were performed in three replicates at each QC level to determine freeze-thaw stability (4 cycles at −70 °C and room temperature), short-term stability (on bench-top at 25 °C for 4 h), post-preparation in dry state (at 2–8 °C for 4 days), autosampler (at 4 °C for 48 h), reinjection stability (at 4 °C for 48 h), and long-term stability (at −70 °C for 77 days). Additionally, stock solution stability was evaluated in different conditions (kept in refrigerator at 2–8 °C for 30 days (sumatriptan), 31 days (terazosin), and on bench-top at 25 °C for 8 h).

### 3.6. Pharmacokinetic and Bioequivalence Studies

Pilot pharmacokinetic and bioequivalence studies of a single dose of 50 mg sumatriptan tablet were performed in six healthy volunteers. The study was designed as an open-label, single dose, fasting, two-treatment, two-period, two-sequence randomized crossover with 7 days washout period between period 1 and period 2 dosing. Subjects were randomly assigned to a treatment sequence (test/reference or reference/test group). Six healthy Thai volunteers with mean age group 32.08 ± 9.20 years and body mass index (BMI) 22.27 ± 1.90 kg/m^2^ were included in the study. Vital signs including pulse (60–90 bpm), blood pressure (SBP of 100–135 mmHg and DBP of 60–90 mmHg), and body temperature (36.5–37.5 °C) were monitored prior to and during the study. All subjects were in good health as shown by clinical laboratory screening including serology, hematology, and biochemistry tests. None of the volunteers reported a history of allergy to sumatriptan, sulfonamides or any of the excipients of this product. All subjects abstained from intake of other drugs and alcohol for two weeks prior to and throughout the study. Caffeine-containing beverages were not allowed in the 7 days prior to and during the study. All the subjects were informed of the aims and risks in the study and written consent was obtained.

In each period, blood samples (6 mL, except 12 mL at 0 h) were collected in tubes containing lithium heparin before (0 h) and at 0.17, 0.33, 0.50, 0.75, 1.00, 1.25, 1.50, 1.75, 2.00, 2.50, 3.00, 3.50, 4.00, 4.50, 5.00, 6.00, 8.00, 10.00, 12.00, and 24.00 h after sumatriptan administration. Plasma samples were separated from blood by centrifugation at 4000 ± 100 rpm, 4 °C for 10 min, aliquoted into microcentrifuge tubes under low light condition, flash frozen immediately and stored at −70 ± 10 °C until analysis. A 15 µL of IS was spiked into a 300 µL of thawed plasma and then followed the procedure described in the sample preparation section.

The pharmacokinetic parameters including the AUC, *C*_max_, *T*_max_, *k*_e_, and *t*_1/2_ were calculated using WinNonlin 6.4 software (Pharsight, NC, USA). Bioequivalence analysis for the two formulations was determined by an analysis of variance (ANOVA) at α = 0.05. Bioequivalence interpretation requires the 90% confident interval (CI) of geometric means test/reference for *C*_max_, AUC_0→t_, and AUC_0→__∞_ to be within the acceptance criteria of 80–125% [22,23]. The study protocol was approved by the Institute for the Development of Human Research Protections (IHRP). The study was carried out in accordance with the international guidelines for human research protection set out by the Declaration of Helsinki, The Belmont Report, CIOMS Guideline, and International Conference on Harmonization in Good Clinical Practice (ICH-GCP).

An incurred sample reanalysis demonstrated the method performance in term of the reproducibility of this assay during BE study. Incurred samples were randomly selected from every volunteer and from every period. The selection criteria included samples which were near the *C*_max_ and the elimination phase in the pharmacokinetic profile of the drug. The results obtained were compared with the data obtained earlier for the same sample using the same procedure. The %different should be within ±20% [24].

## 4. Conclusions

A rapid and convenient LC-MS/MS assay for quantitation of sumatriptan in human plasma was described and validated. This assay employed LLE with *t*-BME, which provided advantages over existing methods in terms of simplicity of extraction procedure, low cost of analysis, appropriate reproducibility, high selectivity, being less-time consuming, and being environment-friendly. The efficiency of LLE and chromatographic run time of 4.0 min per sample allowed the method to be useful in routine analysis. This assay met all US-FDA and EMA guidelines for bioanalytical method validation. The proposed method was applied in the analysis of sumatriptan in human plasma for more than 250 samples per run. The pilot study for the oral administration of sumatriptan 50-mg tablet showed that this developed method could be useful for full bioequivalence study and routine measurement in pharmacokinetic study with a large number of study samples.

## Figures and Tables

**Table 1:** Mean inter-day back-calculated standard and standard curve results (*n* = 3).

Nominal Concentration (ng/mL)	Back-Calculated Concentration (ng/mL)			Mean Back-Calculated Concentration (ng/mL)	%Deviation	%CV
	Day 1	Day 2	Day 3			
0.5	0.51	0.50	0.50	0.50	0.53	0.61
1.0	0.97	1.00	1.01	0.99	−0.97	2.06
2.5	2.57	2.50	2.48	2.52	0.61	1.92
10.0	9.61	9.39	9.19	9.40	−6.01	2.25
20.0	19.52	20.95	20.89	20.45	2.27	3.94
35.0	36.93	35.87	35.70	36.17	3.33	1.84
50.0	50.04	49.58	50.95	50.19	0.38	1.39

**Table 2:** Within-run and between-run accuracy and precision of sumatriptan determination.

Nominal Concentration (ng/mL)	Within-Run (*n* = 6)			Between-Run (*n* = 18)		
	Mean Back-Calculated Concentration (ng/mL)	Accuracy (%Deviation)	Precision (%CV)	Mean Back-Calculated Concentration (ng/mL)	Accuracy (%Deviation)	Precision (%CV)
0.5	0.52	3.23	5.40	0.54	8.30	7.62
1.5	1.42	−5.48	3.91	1.45	−3.60	5.05
25.0	24.85	−0.58	3.93	23.24	−7.05	7.33
45.0	40.33	−10.38	4.43	41.98	−6.71	9.51
50.0	49.94	−0.13	2.15	46.37	−7.27	7.39

**Table 3:** Matrix effect of sumatriptan in nine lots of human plasma (*n* = 3).

Concentration Added (ng/mL)	Plasma Lot	Matrix Factor (%)		IS-Normalized Matrix Effect	%CV
		Sumatriptan	Terazosin (IS)		
1.5	No.1	67.5	88.3	0.765	5.99
	No.2	65.2	86.9	0.751	
	No.3	63.7	85.1	0.748	
	No.4	66.2	86.9	0.761	
	No.5	64.5	85.2	0.756	
	No.6	65.4	83.3	0.785	
	No.7	80.8	90.0	0.898	
	No.8	67.6	86.1	0.785	
	No.9	67.1	88.2	0.760	
45	No.1	79.7	91.4	0.871	1.89
	No.2	78.7	91.6	0.859	
	No.3	77.8	89.0	0.875	
	No.4	77.0	89.2	0.863	
	No.5	74.9	88.1	0.850	
	No.6	80.5	89.6	0.898	
	No.7	84.0	94.2	0.892	
	No.8	80.6	90.3	0.892	
	No.9	79.3	89.9	0.882	

**Table 4:** Hemolytic and lipemic effects of sumatriptan in hemolyzed and hyperlipidemic human plasma, respectively (*n* = 6).

	Nominal Concentration (ng/mL)	Mean Back-Calculated Concentration (ng/mL)	Accuracy (%Deviation)	Precision (%CV)
Hemolytic effect	1.5	1.28	−14.62	6.33
	45.0	43.60	−3.11	2.34
Lipemic effect	1.5	1.28	−14.67	4.26
	45.0	43.07	−4.29	3.65

**Table 5:** Stability results of sumatriptan in human plasma (*n* = 3).

Stability: Storage Condition	Nominal Concentration (ng/mL)	Back-Calculated Concentration (ng/mL)	%Deviation	%CV
Freeze and thaw stability				
After 4th cycle at −70 °C	1.5	1.32	−12.11	6.67
	45.0	39.01	−13.32	4.39
Short-term stability				
Bench-top at 25 °C for 4 h	1.5	1.48	−1.02	9.91
	45.0	41.76	−7.19	1.70
Long-term stability				
77 days at −70 °C	1.5	1.38	−8.27	3.67
	45.0	42.47	−5.62	2.79
Post-preparative stability				
Dry state after extraction at 2–8 °C for 2 days	1.5	1.33	−11.44	3.27
	45.0	38.67	−14.07	2.46
Autosampler at 4 °C for 48 h	1.5	1.64	9.09	6.97
	45.0	49.67	10.37	4.15
Reinjection at 4 °C for 48 h	1.5	1.53	1.84	5.47
	45.0	49.67	6.23	6.43

**Table 6:** Pharmacokinetic parameters for sumatriptan after oral administration of a single dose of 50 mg test and reference products to healthy subjects and 90% confidence intervals of those parameters (*n* = 6).

Pharmacokinetic Parameters	Mean (SD)		Ratio of Least Square Mean T/R (%)	90% Confidence Intervals (T/R)	Intra-Subject CV (%)
	Test Product	Reference Product			
*C*_max_ (ng/mL)	44.08 (14.18)	43.07 (16.40)	103.88	87.18–123.78	9.69
AUC_0→t_ (ng·/mL)	153.07 (45.54)	146.90 (30.80)	102.91	92.68–114.26	14.31
AUC_0→__∞_ (ng·h/mL)	156.95 (46.14)	152.26 (31.74)	101.91	90.47–114.78	8.52
*T*_max_ (h)	1.83 (0.86)	1.46 (0.68)	-	-	-
*t*_1/2_ (h)	2.62 (0.79)	3.42 (2.03)	-	-	-
*k*_e_ (h^−1^)	0.28 (0.08)	0.249 (0.10)			

*C*_max_*=* maximum plasma concentration; AUC_0→t_ = Area under the plasma concentration curve from administration to last observed concentration at time t; AUC_0→∞_ = Area under the plasma concentration curve extrapolated to infinite time; *T*_max_ = time to reach *C*_max_; *t*_1/2_ = half-life; *k*_e_ = elimination rate constant
